# New insights into glial scar formation after spinal cord injury

**DOI:** 10.1007/s00441-021-03477-w

**Published:** 2021-06-02

**Authors:** Amanda Phuong Tran, Philippa Mary Warren, Jerry Silver

**Affiliations:** 1 Center for Integrative Brain Research, Seattle Children’s Research Institute, Seattle, WA USA; 2 Wolfson Centre for Age Related Diseases, Institute of Psychiatry, Psychology and Neuroscience, King’s College London, Guy’s Campus, London Bridge, London, UK; 3 Department of Neurosciences, Case Western Reserve University, Cleveland, OH USA

**Keywords:** Glia, Glial scar, Glial scar formation, Spinal cord injury, Chondroitin sulfate proteoglycans, Single-cell RNA sequencing

## Abstract

Severe spinal cord injury causes permanent loss of function and sensation throughout the body. The trauma causes a multifaceted torrent of pathophysiological processes which ultimately act to form a complex structure, permanently remodeling the cellular architecture and extracellular matrix. This structure is traditionally termed the glial/fibrotic scar. Similar cellular formations occur following stroke, infection, and neurodegenerative diseases of the central nervous system (CNS) signifying their fundamental importance to preservation of function. It is increasingly recognized that the scar performs multiple roles affecting recovery following traumatic injury. Innovative research into the properties of this structure is imperative to the development of treatment strategies to recover motor function and sensation following CNS trauma. In this review, we summarize how the regeneration potential of the CNS alters across phyla and age through formation of scar-like structures. We describe how new insights from next-generation sequencing technologies have yielded a more complex portrait of the molecular mechanisms governing the astrocyte, microglial, and neuronal responses to injury and development, especially of the glial component of the scar. Finally, we discuss possible combinatorial therapeutic approaches centering on scar modulation to restore function after severe CNS injury.

## Introduction

**Fig. 1:**
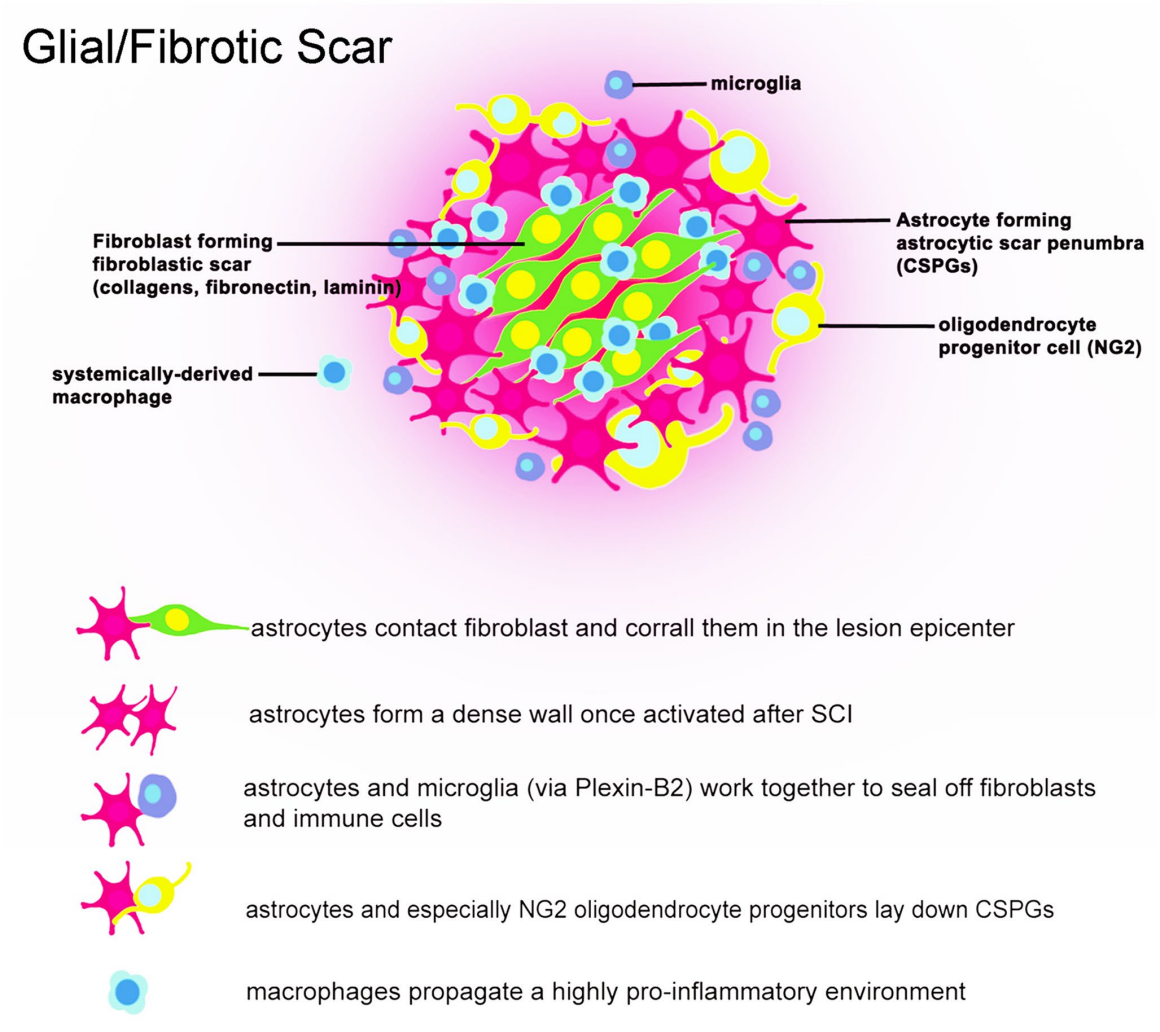
Cellular constituents of the glial/fibrotic scar. Following spinal cord injury, pro-inflammatory cascades activate the multitude of cells found at the spinal cord lesion. The glial scar itself is composed of astrocytes, NG2 + oligodendrocyte progenitor cells, and microglia, among others. Astrocytes and oligodendrocyte progenitor cells of the lesion penumbra are especially important in remodeling the extracellular matrix and upregulating axon-inhibitory chondroitin sulfate proteoglycans (CSPGs). Cells of the lesion penumbra work in concert to cordon off the pro-inflammatory lesion epicenter. The lesion epicenter predominantly includes macrophages and fibroblasts which are sequestered to prevent the spread of inflammation after injury

The failure of repair following spinal cord injury (SCI) is due to factors both intrinsic to cellular components and extrinsic, surrounding the site of injury. In the 1980s, the Aguayo lab first showed that long-distance axon regeneration from CNS neurons within a peripheral nerve graft was abruptly halted at the border between the Schwann cell-laden peripheral nervous system (PNS) and astrocyte-rich central nervous system (CNS) (David and Aguayo 1981). This key finding suggested the importance of extrinsic factors in adversely affecting regeneration following CNS injury. Decades of research have shown that the hypertrophic lesion penumbra which forms the following injury to the CNS (encapsulating reactive astrocytes, activated microglia, and oligodendrocyte progenitor cells) comprises one of three inhibitory cellular compartments which impede axon regeneration both at PNS-to-CNS graft interfaces and within the lesion parenchyma. This is traditionally termed the glial scar (Fig. 1). The second barrier to regeneration, which also resides near the lesion penumbra but lies inside the glial scar, is the fibrotic scar — a structure replete with fibroblasts or fibroblastic-like cells including those derived from the meninges, mural/adventitial sources, or pericytes (Guimarães-Camboa et al. 2017; Riew et al. 2021) (Fig. 1). Swirls of rigid basal lamina membrane form between the astrocytic and fibroblastic layers of the scar further preventing axonal growth (Rudge and Silver 1990; Li et al. 2020b). The third barrier is the lesion epicenter itself, which comprises mostly systemically derived inflammatory cells such as activated macrophages (Busch et al. 2009; Kigerl et al. 2009; Popovich 2014). While all these compartments are dynamic in composition, their formation permanently alters the cellular landscape and extracellular matrix of the spinal cord up to centimeters rostral and caudal from the initial impact. With the accessibility of next-generation sequencing technologies, innovations in SCI research have provided new understanding of the cellular and molecular diversity of cells and structures which encompass the remodeled CNS environment after trauma. This appreciation is stimulating the development of clinically relevant treatment strategies to aid recovery of motor and sensory function.

Here, we review how recent single-cell RNA sequencing and genomic-targeting techniques have expanded our appreciation of the barriers that oppose or promote axon regeneration and functional recovery. This will include a brief review of the differences in regeneration capacity between divergent phyla and neonatal versus adult regeneration after mammalian SCI concentrating on the new insights that emerging techniques reveal about both glia and non-glia in the context of scar formation. We will additionally examine how wound and glial scar-associated products affect neurons. Our review will end with a discussion of the potent growth inhibitory effects of one especially critical family of extracellular matrix molecules, the chondroitin sulfate proteoglycans (CSPGs), and how alleviating these glycoproteins in combination with other strategies can help promote functional recovery.

## Changes in the capacity of CNS regeneration through different phyla and across ages

**Fig. 2:**
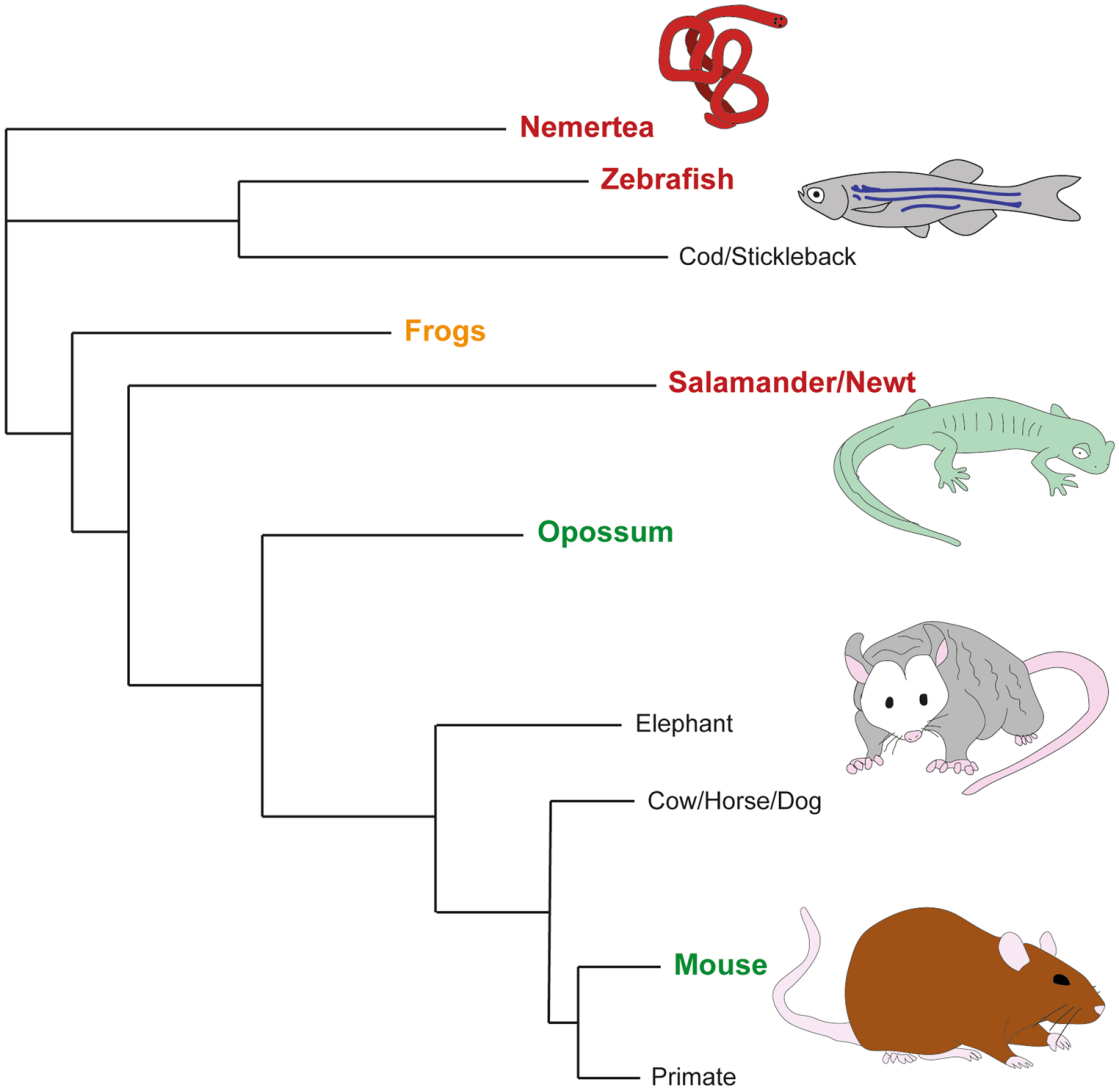
Schematic phylogenetic relationship between regenerating animals. Simplified representation of the phylogenetic relationship between selected species capable of spinal cord regeneration (either within development or through their life span) following injury. Zebrafish, salamanders, and nemertea can regenerate their spinal cords throughout life following injury (red), frogs can do so at the tadpole stage (orange). Some higher-order animals are capable of this in the days following birth (green) including the opossum (until P17) and mouse (until P2). A number of other species (black) with common ancestors to these species are not known to regenerate their spinal cords following injury at any stage of development

Simple invertebrates are capable of regenerating whole-body structures and organs de novo (Fig. 2). Following repeated amputations *Lineus sanguineus* (species ribbon worm, phylum Nemertea) are able to completely regenerate the anterior aspect of their bodies meaning 200,000 worms could be generated following a similar number of dissections, each just 1/200,000th the volume of the original animal (Zattara et al. 2019). However, these remarkable regenerative capabilities are not universal across the ~ 1200 species within the phylum and the defining factors causing this regenerative capacity are not well understood. Zebrafish (*Danio rerio*) (Becker and Becker 2014; Cigliola et al. 2020) efficiently regenerate their spinal cords as adults after injury (Mokalled et al. 2016; Tsata et al. 2020; Klatt Shaw et al. 2021). Among most mature amphibians, complete structural regeneration is lost, but Urodeles (newts and salamanders) regenerate their spinal cords throughout life (Ferretti et al. 2003) (Fig. 2). The ability to regenerate in fish is, in part, due to their capacity for scar-free wound healing (Tsata et al. 2020). The loss of regeneration in amphibians occurs at stages when they begin developing scar-like tissue, likely due to maturation of the immune system (Bertolotti et al. 2013, Edwards-Faret et al. 2021).

**Fig. 3:**
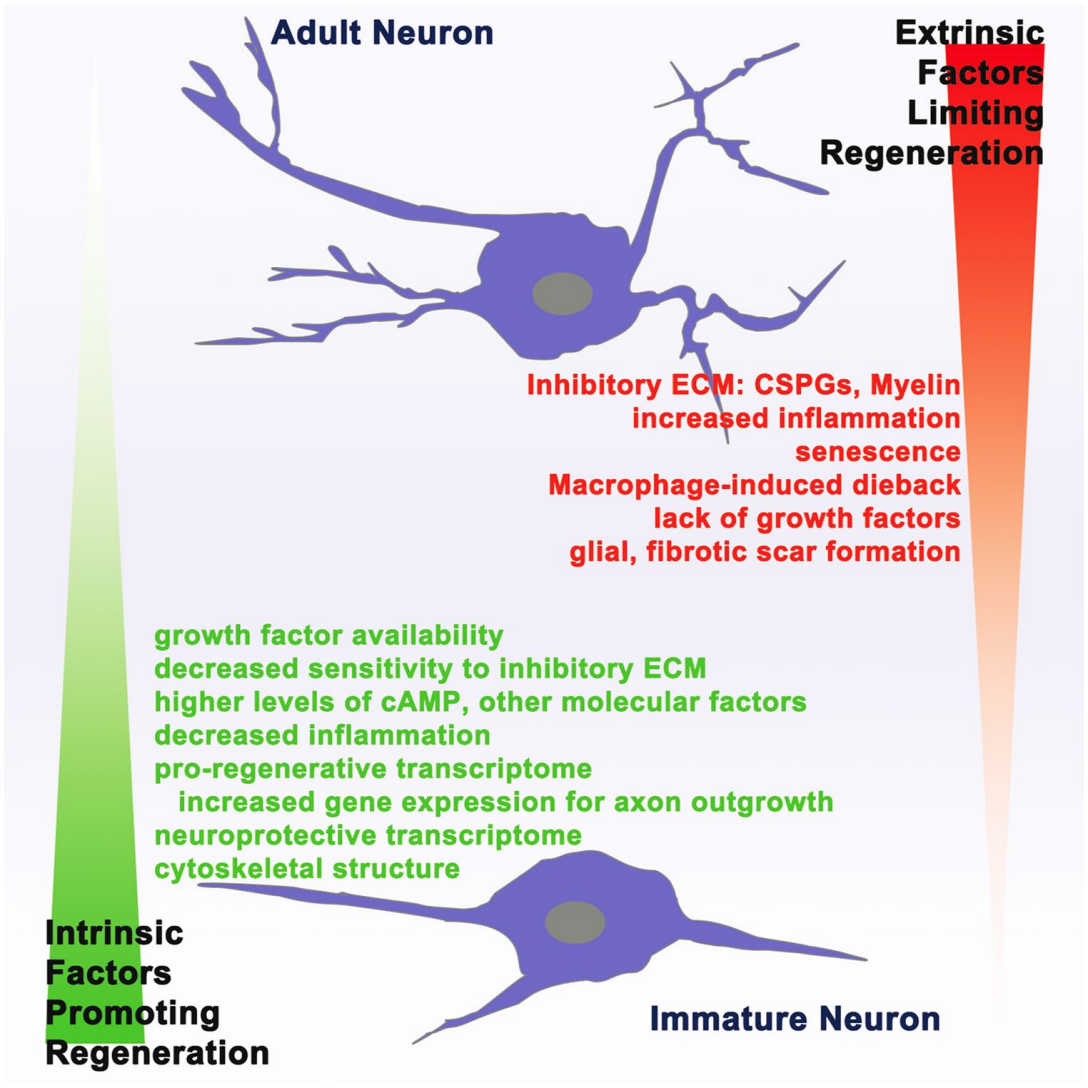
Mammalian neurons lose regeneration potential with age. Embryonic and immature mammalian neurons have a higher potential for axon regeneration after injury due to increased pro-regenerative intrinsic factors and decreased regeneration-inhibitory extrinsic factors compared to the adult mammalian neuron. Immature neurons additionally possess pro-regenerative molecular signals/transcriptome, which is turned off as the neurons age into adulthood

In mammals, the capacity for CNS regeneration sharply declines with age (Fig. 3). This is strikingly illustrated by the South American opossum (*Monodelphis domestica*) which is born precociously*.* Up to 12 days after birth, cervical segments of the opossum cord do not scar (Fig. 2). Regeneration fails rostrally after this time while the lumbar segments, which mature and scar later than the rostral sections, retain the ability to regenerate until 17 days of age (Mladinic and Wintzer 2002). After these developmental stages, scarring occurs globally, and regeneration capacity in the opossum is lost. In mice, the capacity for robust regeneration after birth with scar-free wound healing (following very fine crush injuries to the spinal cord) is limited to the early post-nate (see below sect. “The biology and transcriptomic changes of microglia after injury” for more details). However, following a moderate-to-severe spinal cord transection injury which produces more lesion core-associated inhibitors, even neonatal mice rarely regrow axons, and locomotor and bladder functions never return (Gearhart et al. 1979). Among rats (Sprague-Dawley), robust sprouting (rather than frank regeneration) of the corticospinal tract can occur just after birth, but this capacity for plasticity is lost in the surgically hemisected spinal cord (an extensive lesion) at the end of the first week post-partum (Kunkel-Bagden et al. 1992).

In mammals and birds, the capacity for neurons to regenerate is the greatest during embryonic development (Smith et al. 1987; Steeves and Tetzlaff 1998). For example, neurons derived from embryonic brains transplanted into the more mature CNS are capable of elaborate regrowth and connection-forming abilities (Wictorin and Björklund 1992). Other classic examples of this capacity are shown by Iwashita et al. who implanted embryonic spinal cord grafts into the resected spinal cords of neonatal rats and observed lengthy axonal regeneration with recovery of locomotor function in adulthood (Iwashita et al. 1994). These results were confirmed by Lu et al. who have documented impressive amounts of long-distance regeneration from dissociated neural stem cells harvested from embryonic rats and placed into the spinal cord of adults (Lu et al. 2014). These cells were transplanted in a fibrin matrix impregnated with a host of growth factors to minimize scar and maximize graft integration.

Younger neurons have different responses to extrinsic cues that allow for enhanced axonal regeneration during mammalian development. These include changes in response to inhibitory myelin signaling (Poplawski et al. 2020). Embryonic rat dorsal root ganglion neurons are less sensitive to the inhibitory myelin protein, Nogo-A, whereas by post-natal day 6, the same rat neuron growth cones collapse when exposed to the molecule (Bandtlow 2003). The decreased presence of axon-inhibitory structures such as the scar or perineuronal nets laden with growth inhibitory CSPGs (which do not form until the end of the critical period) plays a vital role (Celio et al. 1998; Takesian and Hensch 2013; Warren et al. 2018a). Specific CSPGs, such as phosphacan, are more highly expressed in the glial scar of the adult compared to the wound response in the neonate (McKeon et al. 1995). It is possible that immature neurons respond differently than adult neurons to CSPGs. Indeed, lack of growth inhibition by CSPGs, as well as their receptors in immature neurons, has been reported (Laabs et al. 2005; Busch et al. 2009; See et al. 2010; Haas and Fischer 2014).

What might be the additional cadre of molecular changes that occur between development and adulthood in the mammal to induce a regeneration-inhibitory “switch?” During development, axons first need to extend toward their intended target. Forward growth then needs to be altered to allow for branching, synaptogenesis, and integration into circuits. This program may, in part, be dictated by interactions between CSPGs and their receptor protein tyrosine phosphatase sigma (PTPRσ) which signal via a newly appreciated relationship with autophagic flux (Sakamoto et al. 2019; Tran et al. 2020). However, the host of transcriptional or molecular switches that allow this change in regeneration capacity with age will surely be multifaceted. Tedeschi et al. hypothesized that genes inhibiting lengthy axon regrowth in the later stages of embryonic development and beyond might be related to mechanisms that control the intrinsic switch from rectilinear growth to that of branching and synapse formation (Tedeschi et al. 2016). Using bulk RNA sequencing, they found that the synaptogenesis-related voltage-gated calcium channel subunit α-2-δ inhibits axon outgrowth at both the end of embryogenesis and throughout adulthood while regulating the differentiation of presynaptic terminals (Kurshan et al. 2009). Blocking the α-2-δ subunit with pregabalin was able to promote CNS (dorsal column) axon regeneration in dorsal root ganglion neurons of adult mice after conditioning sciatic nerve crush lesions. It will be interesting to look for transcription factor(s) and promoter regions that initiate this transcriptomic switch and whether other genes may be involved to cumulatively decrease regeneration capacity with age. These same transcriptomic switches may prove therapeutically beneficial if they can be transiently “turned off” in specific cell populations in adulthood to recapitulate an embryonic state to encourage regeneration.

In addition to the decrease in regeneration capacity from development to adulthood, a growing body of literature suggests that a second, progressive switch occurs between adulthood and old age, further inhibiting regeneration (Geoffroy et al. 2017). One of the cellular mechanisms governing this further regeneration decline may be the scar-enhancing effects of neuroinflammation (Fitch and Silver 2008), which increases in intensity with age (Russo et al. 2011). Chronic neuroinflammation decreases neurogenesis in the hippocampus (Russo et al. 2011) and is responsible for neural stem cell decline in the aging mouse brain (Kalamakis et al. 2019). Stem cell niche-derived cytokines including IFN, CXCL10, and the Wnt agonist, sFRP5, induce a quiescence of stem cells within the subventricular zone. This preserves a small pool of neural stem cells in the aging brain but decreases the amount of neurogenesis compared to younger brains. Work in progress by Paramos-de-Carvalho et al. suggests that, in the aged adult, neurons become senescent following inflammation from SCI and that inhibiting their depletion using a senolytic treatment may improve functional recovery (Paramos-de-Carvalho et al. 2020). Indeed, neuroblast senescence in aged brains increases as it becomes more inflammatory (Jin et al. 2021). Clearly, with age come innate molecular changes based on cellular transcriptional heterogeneity as well as differences in immune responses to injury and exacerbation of inflammation-induced glial scar formation (Fitch and Silver 2008). In the following sections, we will explore how next-generation sequencing technologies with accompanying experimental manipulations are beginning to characterize and define critical molecular differences that lead to regenerative failure in the context of CNS injury or disease with an emphasis on SCI.

## Spinal cord lesions and their cellular constituents

Traumatic SCI begins with physical (primary) injury to the spinal cord, which immediately causes axon shearing, bleeding, and cell death. This leads to the release of alarmins, which recruit local microglia and systemically circulating immune cells that pass through the damaged blood-spinal cord barrier to propagate inflammation and secondary damage (Popovich et al. 1997; Gadani et al. 2015). This ensures that formation of the scar is triggered, in part, by inflammatory processes and requires the concerted effort of a myriad of different cell types as well as transcriptomic and molecular changes driven initially by inflammation-induced reactivity. Here, we will focus on astrocytes, microglia, and neurons. For an in-depth discussion of the fibrotic component of scar, see review by Jae K Lee in this issue.

Next-generation transcript sequencing has emerged as a powerful tool to assess system-wide cellular changes following injury for a broad molecular view of SCI. SCI transcriptomics was assessed as bulk RNA using microarrays and other gene-biased methods. Single-cell RNA sequencing now offers a more sensitive (Chen et al. 2013) and unbiased examination of some RNA transcripts in a variety of cell types to identify novel biomarkers for injury progression, characterize cell heterogeneity/changes after injury, and potentially enhance targeted translation of therapies for SCI. For example, single-cell RNA sequencing revealed that the glial scar penumbra is replete with proliferating oligodendrocyte progenitor cells, which greatly contributes to CSPG deposition and remodeling of the lesion site (Milich et al. 2020). In fact, recent findings suggest that astrocytes, while a major contributor of CSPGs early after injury, are not the predominant CSPG producer over time after SCI (Yang et al. 2020).

However, single-cell RNA sequencing is not without its pitfalls. Rare cell types may be difficult to sequence without specific methods of enrichment for the depth of gene coverage, that is the number of affected genes positively identified in a single cell. The sheer complexity of data from any one experiment makes meaningful interpretation of genomic changes a challenge especially in linking specific gene changes back to biological significance. Added to this, the increased use of this technology among disparate labs with no common naming convention or taxonomy to categorize these newly identified subcellular populations makes collaboration and understanding of the system as a whole difficult moving forward. Nonetheless, recent use of next-generation sequencing has revealed novel insights into cell-specific differences in transcriptomic response after SCI in the following cell types we will focus on here: astrocytes, microglia, and neurons.

### The biology and transcriptomic changes of astrocytes after injury

The regional heterogeneity of astrocytes and their differences in response to injury or pro-inflammatory molecules such as beta-amyloid (Canning et al. 1993) have been further substantiated at the molecular level by next-generation sequencing technologies (Zeisel et al. 2018).

Having established innate molecular diversity among astrocytes, researchers have recently begun to ask how different astrocyte subpopulations respond to injury or inflammation. Huang et al. for example, found that a subset of reactive astrocytes expresses the transcription factors OCT4 and KLF yielding expression of neural stem cell-related markers nestin and sox2 as well as Hippo/Yap pathway activation after SCI (Huang et al. 2020). Overexpression of these transcription factors further conferred improved motor function after SCI in mice. In a further exploration of astrocyte heterogeneity, Zamanian et al. genetically profiled reactive astrocytes termed “A1” derived from mice treated with a systemic LPS injection (Zamanian et al. 2012). Using single-cell microfluidic qPCR, Liddelow et al. found that it was not LPS itself that activated astrocytes (since they do not express TLR4 and MYD88 surface receptors) but rather injury-activated microglia through 1L-1α, TNF, and C1q signaling. Activated A1 astrocytes were found to do the following: upregulate complement cascade genes, were destructive to synapses, showed reduced phagocytic ability, and induced apoptosis in neurons and oligodendrocytes (Zamanian et al. 2012; Liddelow et al. 2017). This is in contrast to “A2” astrocytes, which were described as potentially neuroprotective by upregulating neurotrophic factors (Liddelow et al. 2017). Of note, like the M1/M2 convention of naming reactive macrophages, which has been repeatedly critiqued for its over simplicity (Martinez and Gordon 2014; Ransohoff 2016; Mesquida-Veny et al. 2020), the “A1” and “A2” paradigm, too, flattens the molecular heterogeneity of astrocytes and their diverse response to injury. Thus, while useful, it is important to recall that astrocyte reactivity, as well as their transcriptomic diversity, likely exists on a spectrum as they respond near to or further from a lesion at different ages (Escartin et al. 2021). For example, reactive astrocytes far from a lesion in regions undergoing Wallerian degeneration upregulate GFAP but do not markedly change their orientation, at least within the first few months after injury, and can support robust regeneration even of adult axons (Davies et al. 1999; Li et al. 2020a, b). This is in contrast to astrocytes in the scar surrounding the lesion which upregulate GFAP, undergo hypertrophy, and dramatically change their orientation and density, which ultimately helps the astrocyte block regeneration (Davies et al. 1997; Grimpe et al. 2005; Zukor et al. 2013).

In addition to considerations of innate astrocyte heterogeneity and spatial relationship to the lesion, age is a factor in understanding how astrocytes react to injury. Mature astrocytes transplanted into brains stimulate fibroblast and macrophage entry and enhanced cavitation within (and basal lamina formation around) the lesion site (Smith et al. 1987; Smith and Silver 1988; Filous et al. 2010). Mature astrocytes activated by flow-sorted amyloid plaques upregulate the GABA transmitter, ATP, and glutamate (among others) to impair memory function in mouse models of Alzheimer’s disease (Jo et al. 2014). On the other hand, neonatal astrocytes implanted into the adult, diminished scar formation, and stimulated the regrowth of injured fibers, promoting locomotor function following SCI (Smith et al. 1987; Smith and Silver 1988; Joosten et al. 2004). While immature astrocytes still undergo reactive gliosis following injury (Smith et al. 1987; Balasingam et al. 1994), they do not pack as tightly nor allow for such exuberant basal lamina formation as mature astrocytes. Transcriptional changes to astrocyte precursors after brain stab injury suggest an increase in astrocytic differentiation rather than traditional hypertrophy-associated changes associated with gliosis (Domowicz et al. 2011).

Although astrogliosis occurs in all age groups, the mechanism and potential for glial repair after injury differ greatly between neonatal and adult astrocytes. Neonatal astrocytes secrete neuroprotective factors after ischemic stroke such as PDGF, IGF, and VEGF, which encourage neuronal survival (Wagenaar et al. 2018; Revuelta et al. 2019). Despite their gliotic changes after injury in embryonic and neonatal injuries, transplantation of embryonic astrocytes into the adult lesion site after SCI is a beneficial treatment (Davies et al. 2006, 2011). Various types of neurons seeded onto substrates containing reactive wound tissue harvested from neonatal rat brains (Rudge and Silver 1990; McKeon et al. 1991) extend far longer neurites than those grown on CSPG containing basal lamina-rich wound substrates derived from adult brains. Immature astrocytes were also observed to form bridges between the lesion and tissue border enabling axon outgrowth (Smith and Silver 1988; Filous et al. 2010; Haas et al. 2012). Thus, in contrast to mature astrocytes, especially in the vicinity of older lesions, immature astrocytes are permissive for axon outgrowth.

### The biology and transcriptomic changes of microglia after injury

The roles of microglia and macrophages have often been conflated in SCI research because of the difficulty in distinguishing the two cell populations with immunostaining techniques alone. These two cell populations, however, are distinct. Macrophages, but not microglia, present at the lesion cause dieback of the dystrophic axon tip towards its soma (Horn et al. 2008; Evans et al. 2014). Using a transgenic mouse line that specifically labeled microglia, Bellver-Landete et al. found that microglia proliferated around the lesion site and peaked at 2 weeks post-SCI (Bellver-Landete et al. 2019). This is in contrast to infiltrating monocytes in rats, which peak at 1 week post-injury and again around 60 days post-injury (Popovich et al. 1997; Milich et al. 2020). Activated microglia were also marked by an amoeboid shape and expression of CD68, suggesting increased phagocytic behavior (Janda et al. 2018). By 2 weeks post-injury, microglia serve as a physical barrier between infiltrating leukocytes and astrocytes, which become reactive in response to microglia-derived factors including IGF-1 (Bellver-Landete et al. 2019). These findings suggest that microglia and astrocytes work in concert to seal off infiltrating immune cells and fibroblasts, respectively, to contain inflammation after SCI (Bundesen et al. 2003; Soderblom et al. 2013). Further, microglia-corralling and scar-forming capabilities require an upregulation of the semaphorin receptor Plexin-B2, which peaks by 2 weeks post-injury and wanes by 21 days post-injury to promote motility (Zhou et al. 2020). Depleting microglia with a stimulating growth factor receptor antagonist, PLX5622 (Bellver-Landete et al. 2019; Fu et al. 2020), or conditionally knocking out Plexin-B2 specifically in microglia (Zhou et al. 2020) resulted in an increase in systemic immune cell infiltration, increased neuronal and oligodendrocyte apoptosis, disruption of glial scar formation primarily through disorganized and delayed astrocyte repopulation, and worsened behavioral outcomes after SCI. Therefore, in adulthood, microglia work together with other immune cells such as macrophages and other glia (including astrocytes) to aid in effective scar formation. Recent findings have even revealed that astrocytes and microglia coordinate to maintain brain homeostasis by working in concert to phagocytose specified territories of apoptosed neurons (Damisah et al. 2020). The ability of microglia and astrocytes to efficiently clear apoptosed cells and other debris declines with age (Damisah et al. 2020), which could further inflammatory processes over time. Our current understanding corroborates that glial scar formation is an immediately beneficial process, comprising many cell types (beyond that which is discussed here) to contain and limit the spread of inflammation. However, over time, the adult scar and its extracellular matrix components impede regeneration (see below and (Tran et al. 2018b)).

While microglia were shown to coordinate scar formation during adulthood, a recent seminal study by Li et al. highlights the difference between microglia-driven scar-forming, regeneration blocking, wound healing in the adult and scar-free, and regeneration promoting healing in the neonate (Li et al. 2020b). Li et al. performed narrow spinal crush injuries in neonatal (post-natal day 2) mice, which led to a lesion-associated increase of loosely organized GFAP-positive astrocytes, but with little obvious hypertrophy and with an absence of classic adult scar-associated matrix constituents. This contributed to wound healing without scar formation and allowed for robust growth of serotonergic and cortico-spinal axons directly through the lesion. Notably, axon growth largely failed to extend past the injury site when lesions were made slightly later (by post-natal day 7), or in the adult. While the authors could not determine whether axon growth was due to regeneration from injured cells or the arrival of late projecting fibers, it was clear that the scar-free wound healing response in the neonate allows for axonal growth into and well beyond the lesion. Importantly, there was total absence of an adult-like scar with little deposition of CD68 + immune cells or basal lamina constituents such as fibronectin, collagen type I, laminin, or CSPGs. It was further demonstrated that axon growth-blocking glial scar formation of the post-injury neonatal spinal cord was critically dependent on immature microglia as their depletion through conditional knock down, or treatment with a drug to inhibit colony-stimulating factor 1 receptor allowed for much stronger astrocytic hypertrophy and packing density. These treatments dramatically reduced but did not completely eliminate, the efficacy of axon growth into or through the lesion site. The vast majority of failed regenerating serotonergic axons now halted their growth directly abutting the dense astrocytic wall, revealing clear evidence that astrocytes (likely in and of themselves) can build a regeneration-blocking scar barrier whose inhibitory properties at this stage appear to arise largely from the mere physical arrangement and packing density of the cells (see (Xu et al. 1999) for the role of GFAP in the compaction of scar astrocytes). Importantly, the lack of potently inhibitory basal lamina constituents such as collagens and CSPGs likely allowed for a small number of neonatal axons (with their enhanced growth machinery compared to adult neurons) to pass through the barrier.

Using single-cell RNA sequencing, Li et al. found five transcriptionally distinct microglia populations after injury, confirming a heterogeneous response. Further analysis suggested that neonatal microglia become transiently reactive after injury, then revert to a homeostatic state by 3 days post-injury (Li et al. 2020b). As part of this transiently reactive response, neonatal injury-activated microglia first secreted fibronectin and remodeled the extracellular matrix to close the wound and enable an axon growth-permissive bridge. This vital response to enable regeneration is curtailed in the adult where fluid-filled cysts and the glial scar form (Rooney et al. 2009). The initiation of inflammation and engagement of the CNS immune system to propagate regenerative processes also bring to mind Ernst Haeckel’s adage that “ontogeny recapitulates phylogeny” as the zebrafish regeneration response system also relies on an initial inflammatory signal to start neural stem cell proliferation and regeneration after a brain injury or SCI (Kyritsis et al. 2012). Increasingly, recent findings are highlighting a major difference in injury responses between aged and immature immune cells. Young neutrophils (Ly6G^lo^), characterized after being activated by zymosan, were found to drive retinal ganglion cell axon regeneration by secreting growth factors such as NGF and IGF-1 (Sas et al. 2020). Transplantation of this cell type into the sciatic nerve enabled dorsal root ganglion axon regeneration into the lacerated dorsal column of the thoracic spinal cord (Sas et al. 2020). Immature microglial cell transplantation into the injured adult spinal cord also improved wound healing without scar and permitted axonal regeneration beyond the lesion (Li et al. 2020b).

Li et al. (2020a, b) emphasize the importance of proteases in scar formation. In the neonate, microglia produce endogenous proteinase inhibitors to promote healing. They found that serine protease inhibitors and cystein peptidases such as *Cstb* encoding cystatin b, which inhibits lysosomally derived cathepsin proteases, were upregulated (Li et al. 2020a, b). Blocking proteinases with inhibitors prior to transplanting mature microglia into the lesioned adult cord led to reductions in collagen I and CSPG deposition which, in turn, resulted in some regenerating axons, although fewer than that which occurred after transplanting purified immature microglia. This suggests the likely existence of other scar-free wound healing factors. Rampant protease secretion from immune cells propagates their entry through the blood-brain barrier after traumatic injury (Noble et al. 2002) or neurodegenerative disorders (Crapser et al. 2020) contributing to secondary damage and loss of synapses. However, the fine control of local protease release from growth cones or the leading processes of migrating cells is critically important during developmental pathfinding (Brooks et al. 2013) as well as during regeneration or sprouting in the adult (Krystosek and Seeds 1984; Luo et al. 2018; Tran et al. 2018a; Carulli and Verhaagen 2021; Tran and Silver 2021).

### The response of neurons after SCI

At the acute stage following injury, sheared axons undergo a process called dieback that drives their projections back towards the soma. Axons at the lesion epicenter can be cut by the shear force of traumatic SCI as well as massively damaged by invading macrophages (Fitch et al. 1999). In response to injury, severed axons die back from the lesion epicenter upon physical contact with infiltrating, reactive macrophages (Horn et al. 2008; Busch et al. 2009). This process is dependent on macrophages directly contacting axons and the subsequent secretion of proteases such as MMP-9 (Busch et al. 2009, 2011). Treatment of macrophages with MMP-9 specific inhibitors, anti-inflammatory drugs, or other inflammatory-modulating substances was able to greatly reduce the macrophage-induced dieback phenomenon (Busch et al. 2011; DePaul et al. 2015). Thus, dieback and ensuing axon damage are a consequence of the pro-inflammatory environment shortly after SCI. Interestingly, embryonic neurons are resistant to attack by activated macrophages (Busch et al. 2009).

In response to the formation of the glial scar, the growth cone tips under attack by macrophages eventually cease retracting and become “entrapped” in the reactive astrocyte/OPC/pericyte penumbra of the lesion (Davies et al. 1997; Filous et al. 2014; Son 2015) where they can reside in a stable, dystrophic state for decades (Ruschel et al. 2015). These growth cones, trapped indefinitely in the glial scar penumbra, may be able to do so because they develop synaptic-like contacts on CSPG producing (NG2) glia that colocalize with synaptic markers such as PSD95 and SNAP25 (see below and (Filous et al. 2014)).

### Transcriptomic changes of neurons after SCI

Next-generation sequencing has revealed a greater diversity of spinal cord neurons than previously considered. Sathyamurthy et al. created an atlas of the adult mouse lumbar spinal cord and identified more than 40 distinct neuronal subpopulations (Sathyamurthy et al. 2018). Of note, five neuronal clusters of the ventral horn and one along the mid-cord expressed the perineuronal net CSPGs aggrecan and brevican (Sathyamurthy et al. 2018), showing that perineuronal nets surround previously unidentified ventral neurons (Galtrey et al. 2008). The molecular diversity of lumbar spinal cord neurons is surprisingly heterogeneous — this may also be the case with neurons of other regions of the spinal cord, but how and which important molecular differences underpin their unique responses to SCI or scarring are yet to be determined. For example, SCI researchers have long observed that, within the CNS, serotonergic neurons respond most robustly to various forms of therapeutic treatments, are less likely to die back due to macrophage attack, and are most likely to sprout following CNS lesions (Li et al. 2004; Pearse et al. 2004; Alilain 2009; Alilain et al. 2011; Hawthorne et al. 2011; Lang et al. 2015; Jin et al. 2016; Warren et al. 2018b). Propriospinal interneurons have also been reported to regenerate their axons directly through the early lesion environment after thin midline cuts to the spinal cord (Fenrich and Rose 2009). In contrast, a classic example of a regeneration-refractory and poorly sprouting pathway is the corticospinal tract, an important system involved in voluntary motor control (Welniarz et al. 2017).

Recent studies revealed insights into the growth characteristics of the corticospinal tract, which matures relatively late in development with elongation and synaptogenesis continuing post-natally (Bates and Stelzner 1993). In rodents, late-arriving corticospinal axons can circumnavigate lesions during the first post-natal week (Schreyer and Jones 1983). However, regeneration of this tract in the adult does not occur. To better understand this tract’s unique response following SCI, Tsujioka et al. performed a unilateral pyramidotomy to track changes and compared gene expression of the cervical cord between 7-day-old and 8-week-old mice at 3 days post-pyramidotomy (Tsujioka and Yamashita 2019). Tsujioka et al. found that the inflammatory response was different between neonatal and adult mice: adults showed an increase of Iba1 + microglia/macrophages near the denervated tract after pyramidotomy whereas neonatal mice did not show any such long-term accumulation (see also Li et al. 2020b). The neonatal group also increased gene networks responsible for axon regeneration, myelination, and cell proliferation. In the adult, Tsujioka et al. found enrichment of genes involved in lysosomal activity and phagocytosis, toll-like receptor signaling, and coagulation and complement pathways by microglia/macrophages. Adults showed high inflammatory response activation marked by Ccl5 and Cd52 and an increase in cell proliferation and cell cycle-regulating genes. While this study identified which genes were up- or downregulated following injury, the dosage or extent of gene expression especially between the two different age groups when genes themselves may be innately subject to changes in activation or downregulation could not be assessed. This caveat, however, could be addressed by comparing the transcriptome of the same age group under different conditions such as with a control, non-regenerating group, and a treatment group where axon regeneration is encouraged.

In this regard, Poplawski et al. (2020) performed a dorsal column lesion at the cervical level to transect the corticospinal tract in adult mice. In one group, neural progenitor cells derived from embryonic day 12 mice (which had been shown to allow for corticospinal tract regeneration and synapse reformation Lu et al. 2012, 2014)) were grafted into the lesion, and mRNAs enriched from corticospinal neurons were subjected to single-cell RNA sequencing at 10, 14, and 21 days after injury. A remarkably pro-regenerative gene network similar to an embryonic transcriptional growth state (embryonic day 18 corticospinal neurons) was initiated by injury alone, although it disappeared as soon as 2 weeks following injury. However, animals with neural progenitor cell grafts were able to sustain this pro-regenerative response. In cases of successful corticospinal axon regeneration, it was found that there were increases in both the neuroprotective genes early after injury and the expression of genes responsible for inducing synapse formation and axon guidance. Notably, genes such as *Htt* encoding the Huntington protein (Htt), which aids embryo survival and selective autophagy (Rui et al. 2015), were identified as essential to this pro-regenerative response. The discovery of the Htt protein as a component of axon regeneration (Poplawski et al. 2020) further pinpoints the importance of autophagy as one of the many processes essential to axon regeneration (Tran et al. 2020). Interestingly, upregulation of this embryonic response is sustained by neural progenitor cell grafts instead of recruiting other genes not formally part of the embryonic response. Thus, recapitulation of an embryonic response and maintenance of this response may be a meaningful therapeutic approach moving forward.

## SCI and plasticity of the glial scar

The glial/fibroblastic scar forms from the synergistic interactions of astrocytes and microglia in cooperation with several additional cell types including, but not limited to macrophages, oligodendrocyte progenitor cells, pericytes/fibroblasts, ependymal (Meletis et al. 2008), and endothelial cells. These cells work in tandem under a highly inflammatory environment following traumatic SCI to proliferate, activate, and cordon off further inflammatory propagation from the lesion epicenter (Tran et al. 2018b). The remarkable increase in density of the scar-forming reactive astroglia that (after many months) eventually fill the territory left after removal of cell debris (Silver and Miller 2004) as well as the remodeled extracellular matrix (in particular the basal lamina constituents, CSPGs, and collagens) strongly inhibit the regeneration of axons (Davies et al. 1997; Bradbury et al. 2002; Hara et al. 2017; Dias et al. 2018). However, in very special circumstances (after thin lesions that limit basal lamina production or lesion core inflammation or during the early formative stages of scar development), the astrocyte component of scar is not an absolute barrier and, if they bridge the lesion, can even allow for some regeneration when combinations of strongly intrinsic growth-promoting and/or extrinsic inhibitory factors are modulated (Rudge and Silver 1990; McKeon et al. 1995; Liu et al. 2010; Zukor et al. 2013; Anderson et al. 2016; Silver 2016).

Recent work further contextualizes this view characterizing key differences between the subacute (2 weeks) and chronic (8 weeks) post-SCI glial scar (Li et al. 2020a). Tandem mass tag-based quantitative proteomic analysis of the glial scar secretome was performed over time in a model of complete transected thoracic SCI in Sprague-Dawley rats. The subacute glial scar secreted up to three times the amount of growth factors such as bFGF, VEGF, and PDGF compared to the chronic scar. As previously shown (Andrews et al. 2012; Yi et al. 2012), the expression of CSPGs in the lesion penumbra changes with time, with at least four times the amount of detected CSPG-glycosaminoglycans (GAGs) forming in the chronic than subacute glial scar. Among other axon-inhibitory extracellular matrix proteins, Hara and colleagues found that collagens (Hara et al. 2017) were also upregulated in the chronic scar. Pharmacological blockade of the interaction between reactive astrocytes and type I collagen prevented glial scar formation, allowing for a more loosely arranged glial architecture which, in turn, aided axonal regrowth and functional recovery following SCI in the mouse (Hara et al. 2017). Importantly, surgical removal of the chronic scar (8 weeks post-injury) produced significant ascending sensory tract axons regenerating into the lesion site with the aid of BDNF as assessed by cholera toxin B tracing. These were the same neuronal subtypes identified, and techniques used in research as failing to regenerate following acute astrocyte ablation which releases the highly inhibitory early lesion core inflammatory population (Anderson et al. 2016; Silver 2016). Thus, while the subacute astroglial scar is important in wound healing and corralling inflammatory processes, it contributes to (but is not the only constituent of) the chronic impediment to axon regeneration. The glial scar should be viewed as a dynamic, time-dependent, and constantly remodeling structure. Reactive and scar astrocytes can, for a time, be highly plastic cells whose growth supportive or inhibitory functions are determined by local signaling events (DePaul et al. 2017). For example, grafting skin precursor-derived Schwann cells into a chronically contused spinal cord can alter the formation of the extremely dense and molecularly inhibitory, barrier-forming scar at the astroglial/Schwann cell interfaces through stimulating the astrocytes to decrease inhibitory ECM secretion, re-orient, and migrate well into the graft (Assinck et al. 2020). This dynamic action allowed for the regeneration of catecholaminergic axons into and beyond the lesion epicenter.

### CSPGs impede regeneration/plasticity

**Fig. 4:**
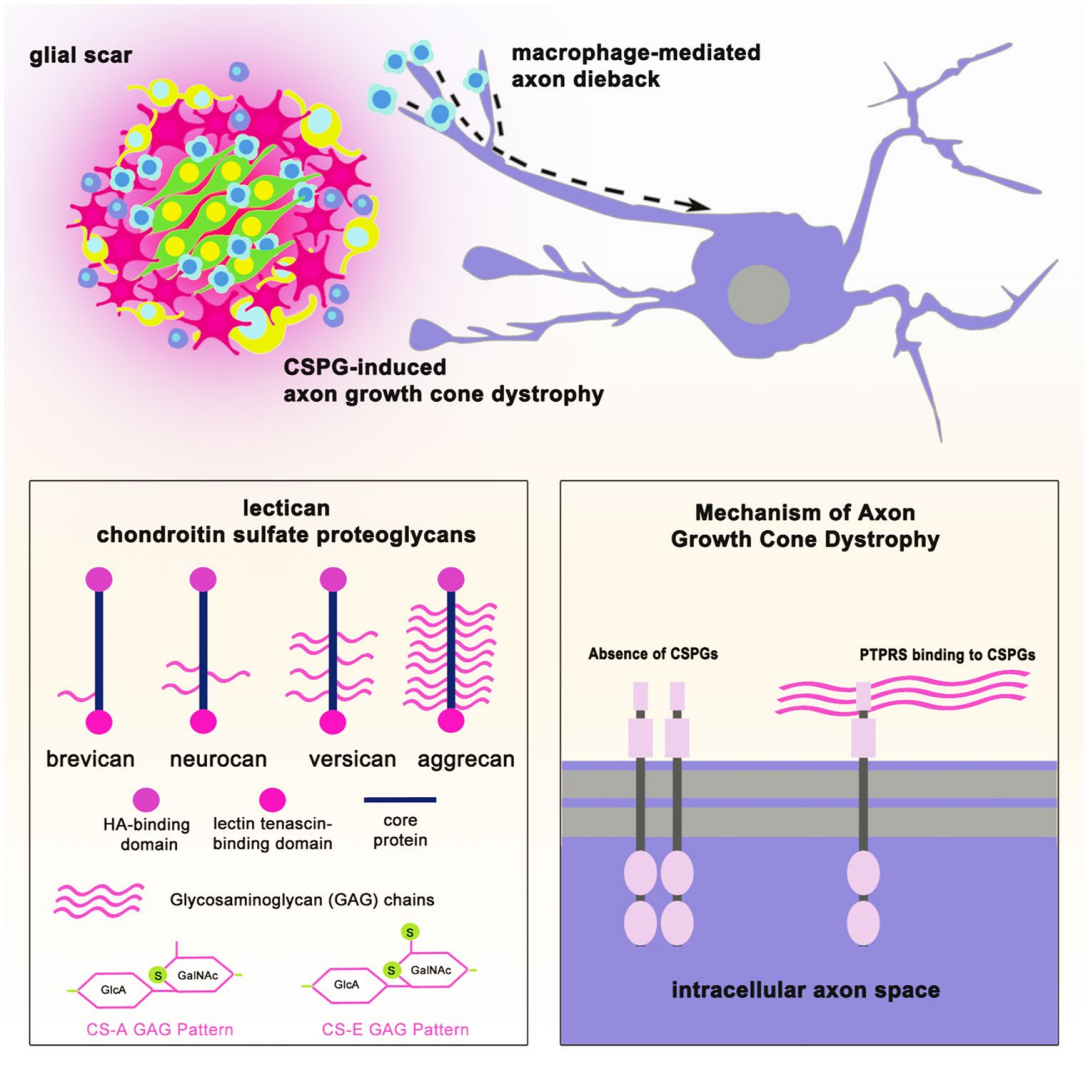
Mechanisms of axon regeneration failure. Acutely after spinal cord injury, infiltrating macrophages come into physical contact with the tips of sheared axons causing the injured axon to dieback to the neuron soma. Chronically, chondroitin sulfate proteoglycans (CSPGs) of the glial scar cause growth cone dystrophy of approaching axons. CSPGs consist of a lectican group (brevican, neurocan, versican, and aggrecan) and phophocan and NG2 (not pictured here). The glycosaminoglycan (GAG) chains, notably sulfation patterns CS-A and CS-E, are especially inhibitory to axon regeneration. GAG chains of CSPGs bind to protein tyrosine phosphatase receptor sigma (PTPRS) promoting monomerization of the receptor at the axon growth cone to cause dystrophy and chronic regeneration failure

A key inhibitory component of the glial scar are the CSPGs, secreted early on by reactive astrocytes and in the chronic phase by various cell types (Tran et al. 2018b). Other extracellular matrix proteins of the chronic glial scar (collagens, tenascins, semaphorins, and ephrins) additionally provide inhibition to regeneration following SCI (Tran et al. 2018b). CSPGs are extracellular matrix proteins composed of a protein core and a varying number of sugar moieties called glycosaminoglycan (GAG) chains (Fig. 4). They include three major subtypes: secreted lecticans, membrane-bound phosphacan (PTPRZ1), and NG2 (CSPG4).

The lecticans are also found endogenously in specialized extracellular matrix structures called perineuronal nets (PNNs), which surround the soma and proximal dendrites of certain subpopulations of neurons including fast-spiking parvalbumin and GABAergic neurons (Fawcett et al. 2019). During development, PNN formation closes the critical period to ensure neural circuit stability (e.g., in the visual pathway) (Liu et al. 2013). In the spinal cord, the majority of PNNs are found in the ventral motor horn (Irvine and Kwok 2018). Circuit stability is conferred by CSPGs (Warren and Alilian 2018; Warren et al. 2018a; Carulli and Verhaagen 2021). Perhaps through dampening autophagic flux (Tran et al. 2020), CSPGs through protein tyrosine phosphatase receptor sigma (PTPRσ) binding promote initial adhesion and receptor recruitment necessary for synaptogenesis (Han et al. 2016; Bomkamp et al. 2019). It has recently been found that the PNN-PTPRσ complex exerts its inhibitory action on neuronal plasticity, at least in part, by restricting signaling of the neurotrophic receptor tyrosine kinase 2 (TRKB) (Lesnikova et al. 2021).

### The LAR family receptors mediate the inhibitory actions of CSPGs

Leukocyte common antigen-related tyrosine phosphatase receptor (LAR) (Fisher et al. 2011) and PTPRσ (Shen et al. 2009) both confer inhibitory CSPG signaling (Fig. 4). The CS-E motif of CSPGs through PTPRσ binding dampens autophagic flux (Martin et al. 2011) through dephosphorylation of the actin cytoskeleton-binding protein cortactin, perturbing autophagosome and lysosomal fusion (Sakamoto et al. 2019). By imposing autophagic dysfunction, especially in the context of the post-SCI environment, CSPGs induce axon growth cone dystrophy and axon regeneration failure (Tom et al. 2004; Sakamoto et al. 2019; Tran et al. 2020). Notably, PTPRσ is a bifunctional receptor in that both axon-inhibitory CSPGs and axon-promoting heparan sulfate proteoglycans (HSPGs) bind (Coles et al. 2011, 2014). CSPG-PTPRσ binding promotes monomerization of the receptor to increase its dephosphorylation activity, while HSPG-PTPRσ binding promotes clustering of the receptor and a decrease in dephosphorylating activity (Wu et al. 2017). In the extracellular matrix milieu, the attention of the growth cone to the ratio of HSPGs to CSPGs as well as other extracellular proteins such as reelin (Zluhan et al. 2020) or laminin (McKeon et al. 1995) is, therefore, important in determining whether axon outgrowth proceeds. Following SCI, however, approaching axons are strongly inhibited by the preponderance of upregulated CSPGs found radiating from the glial scar (Davies et al. 1997). Importantly, the interaction of CSPGs with PTPRσ causes axonal growth cones to enter an entrapped dystrophic state, preventing regeneration after SCI not through repulsion but, rather, through an overly adhesive mechanism (Lang et al. 2015).

### Perturbing CSPG inhibition promotes regeneration/sprouting and functional recovery

Directly cleaving GAG chains from CSPGs using chondroitinase ABC has proven effective in restoring plasticity, axon regeneration/sprouting, and functional recovery following SCI (Bradbury et al. 2002; Alilain et al. 2011; Warren et al. 2018b, Warren et al. 2021). Perturbing CSPG signaling through PTPRσ is an another effective strategy to enhance regeneration of axons and myelin. Pleiotrophin binding to glypican-2 forms a complex with CSPGs to abrogate CSPG binding to PTPRσ (Paveliev et al. 2016). Enoxaparin has recently been shown to promote functional recovery after SCI by antagonizing PTPRσ (Ito et al. 2021). Our own work in synthesizing a peptide, ISP (intracellular sigma peptide), modeled against the regulatory wedge domain of PTPRσ to “turn off” CSPG-PTPRσ signaling resulted in the rescue of the axon growth cone from becoming entrapped and dystrophic and remarkably enhanced bladder and coordinated locomotor recovery following systemic application in rat models of contusive SCI (Lang et al. 2015; Rink et al. 2018). Treatment of dorsal root ganglion neurons and oligodendrocyte progenitor cells (Luo et al. 2018; Tran et al. 2018a) with ISP-induced autologous localized protease release, which was capable of immediate degradation of CSPGs to enhance process outgrowth or maturation and cell survival, respectively. In dorsal root ganglion neurons, ISP-induced focal release of the lysosomal protease, cathepsin B, was effective in digesting CSPGs located around the advancing growth cone to encourage outgrowth past a CSPG gradient (Tran et al. 2018a). In oligodendrocyte progenitor cells, the ISP-induced protease was MMP-2, which also relieved CSPG-inhibition through degradation, encouraged digestion of CSPGs in demyelinated lesions, and promoted remyelination through restoration of oligodendrocyte migration, maturation, and homeostasis (Luo et al. 2018; Tran et al. 2018a). In both cell types, cell migration was increased. This may in part be through due to disruption of CSPG-PTPRσ signaling which dampens autophagy (Sakamoto et al. 2019; Tran et al. 2020).

During development, increases in cytoskeletal stability are typically associated with differentiation of an axonal rather than dendritic phenotype (Witte et al. 2008). It has been shown that further increasing microtubule stability in the adult can promote axonal regeneration after traumatic SCI (Hellal et al. 2011; Ruschel et al. 2015; Ruschel and Bradke 2018). Interestingly, administration of tubulin stabilizing pharmaceutical therapies (taxol and epothilone B and D) also acts to rearrange the cytoskeleton of surrounding astrocytes at the site of injury, reducing CSPG secretion and thus facilitating axonal growth (Hellal et al. 2011). It is now important to determine how and where in the dynamic microtubules of the cytoskeleton this stabilizing effect occurs to promote this duel neuronal and glial effect facilitating long-range transport (Ertürk et al. 2007) and alteration of the extracellular matrix.

### CSPGs impact on the immune system

CSPGs contribute to proinflammatory signaling in immune cells through the CD44 receptor, which results in enhanced TNFα secretion by microglia/macrophages (Rolls et al. 2008). This further prolongs the post-SCI inflammatory environment and secondary damage to tissue. Depletion of GAGs with chondroitinase ABC is able to encourage microglia/macrophages toward an alternative inflammatory phenotype, which conferred neuroprotection and improved recovery after acute SCI (Bartus et al. 2014; Didangelos et al. 2016). So, too, in a mouse model of inflammatory multiple sclerosis, experimental autoimmune encephalomyelitis (EAE), CSPGs exacerbate cell-damaging inflammatory processes by stimulating macrophages to produce proinflammatory cytokines (Stephenson et al. 2018). Analysis of a multiple sclerosis genome-wide associated screen led to the discovery of the glycosyltransferase exotosin-like 2 gene, which normally limits the cellular production of CSPGs. Knockout of this gene resulted in an increase of CSPG deposition within lysolecithin-induced focal demyelinated lesions (Pu et al. 2020). This resulted in the increased recruitment and activation of microglia/macrophages to demyelinated lesions and further axon loss. Importantly, reduction of CSPGs in mouse models of demyelination through inhibition of its synthesis (Keough et al. 2016) or by enhancing oligodendrocyte maturation and autologous production of CSPG-degrading proteases via the use of ISP (Luo et al. 2018) encouraged regeneration of myelin and functional recovery.

## Combinatorial strategies to promote functional recovery after SCI

Emphasis on the multitude of cells involved in the formation of the glial/fibrotic scar highlights the many hurdles required for post-SCI regeneration and ultimately functional recovery. These obstacles, for the most part, can be encompassed by two problems: an intrinsic one whereby the adult neuron lacks a robust “motor” for axon process outgrowth compared to their immature counterparts (Zukor et al. 2013; Andrews et al. 2016; Cheah et al. 2016; Fawcett 2017), and an extrinsic one where the adult spinal cord suffers from a dearth of substrate adhesion molecules or their receptors while the remodeled chronic SCI environment is replete with inhibitory extracellular matrix proteins. Moving forward, combinatorial strategies that address these major obstacles will be necessary to best encourage functional recovery. In a striking example of the challenges present in restoring functional recovery after injury, Bei et al. illustrated how promoting axon regeneration through boosting retinal ganglion neurons’ intrinsic growth motor using a PTEN and SOCS3 co-deletion is itself insufficient after optic tract transection as conductance of de novo; unmyelinated axons were lacking (Bei et al. 2016). However, PTEN and SOCS3 co-deletions in combination with myelin-promoting growth factors OPN, IGF1, and CNTF improved optomotor function. Still, other studies have sought to recapitulate developmental programs enabling axon growth (Filbin 2006; Lee et al. 2013; Hilton and Bradke 2017; Courtine and Sofroniew 2019).

## Conclusions

In the past decades, our understanding of the glial scar has grown to encompass much more than reactive astrocytes around the lesion penumbra. We currently appreciate that the glial/fibrotic scar comprises a myriad of cells including (but not limited to) macrophages, microglia, oligodendrocyte progenitor cells, pericytes/fibroblasts, ependymal cells, and endothelial cells, with neuron growth cones entrapped in the lesion penumbra. Here, we have discussed how next-generation sequencing technologies have revealed how complicated this post-injury structure is at the cellular, molecular, and transcriptomic levels with an emphasis on the pre- and post-astrocytic, microglial, and neuronal cellular heterogeneity. Acutely, this structure is indispensable to the wound healing process to limit further secondary tissue damage and to resolve rampant inflammatory processes. Chronically, however, the typical glial/fibrotic scar that forms after contusive or extensive surgical lesions with a balance of extracellular matrix proteins tipped in the direction of inhibition, including CSPGs, poses a hugely inhibitory biochemical and physical barrier to successful axon regeneration and functional recovery. Our understanding of the uniquely heterogeneous composition of the scar and its evolution over time advocates for a combinatorial strategy to encourage axon regeneration and functional recovery. Treatment strategies that simultaneously target both the intrinsic problem of decreased axon outgrowth machinery as well as the extrinsic hurdles of increased axon-inhibitory proteins coupled with targeted rehabilitation are our best options going forward.
